# Monotonic and Creep Studies on the Pull-Through Resistance of Laminated Glass with Locally Embedded Steel Mesh

**DOI:** 10.3390/ma15207083

**Published:** 2022-10-12

**Authors:** Marcin Kozłowski, Dominik Wasik, Kinga Zemła

**Affiliations:** Department of Structural Engineering, Silesian University of Technology, Akademicka 5, 44-100 Gliwice, Poland

**Keywords:** laminated glass, point-fixing, monotonic, reinforcement, steel mesh, creep, pull-through resistance, experiments, numerical study

## Abstract

The paper deals with the phenomenon of post-breakage capacity in point-fixed laminated glass elements and reports the results of an ongoing research project aimed at developing a reinforced point-fixed laminated glass element with locally embedded steel mesh and increased post-breakage capacity. The work involved monotonic and creep studies on the pull-through resistance in a custom-made experimental setup. A total of 12 test series and 48 specimens were tested, including reference and reinforced samples. In the monotonic loading experiment, the load increase after the initial glass breakage was observed for all specimens. However, the reinforced specimens with embedded steel mesh showed significantly improved post-breakage capacity. It was found that the local reinforcement in the direct vicinity of the hole in the glass increased the post-breakage strength by 46.3%, 102.6%, and 156.2% for reinforcement diameters of 75 mm, 110 mm, and 150 mm, respectively. Moreover, the creep study found that the reinforcement significantly increased the time to failure.

## 1. Introduction

**Figure 1:**
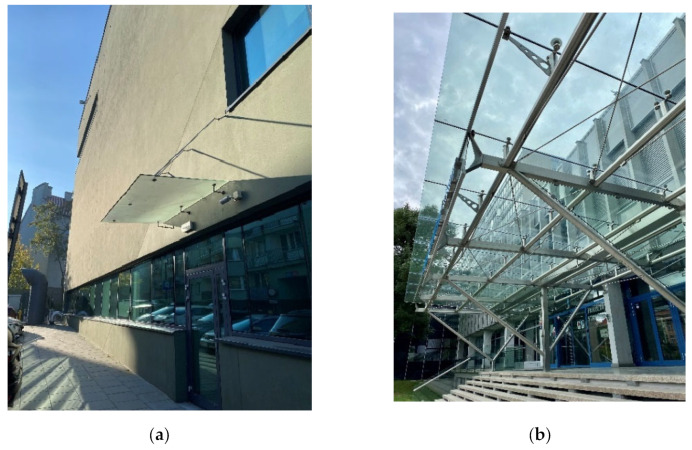
Examples of point-fixed laminated glazing: (**a**) a canopy with fixing rods mounted above a building’s entrance; (**b**) glass infill panels mounted to a steel structure (pictures by M. Kozłowski).

Glass has recently become an important material in modern architectural applications [1,2]. Its thermal, energy, light, and aesthetic attributes have led to the continuously increasing use of structural glass and the evolution of its design toward safe, geometrically complex, and robust mechanical systems [3]. Glass shows high stiffness, significant compressive strength, and environmental durability. However, unlike traditional building materials, it shows inherently brittle behaviour and, thus, cannot redistribute stress concentrations via plastic deformation [4,5]. When overloaded, glass fractures within milliseconds without warning signs of impending cracking; thus, its failure can be sudden and catastrophic. Therefore, the fail-safe approach is commonly used to design structural glazing [6,7,8]. In the event of a fractured state, an element must preserve a residual stiffness and load-bearing capacity to maintain resistance under permanent loads and a certain level of variable loads [7]. The post-breakage capacity can be provided by the redundancy of the glass component, undamaged plies of that glass component, or structural alternative load paths [6]. This demand excludes the use of monolithic glass as a structural material, whereas laminated glass—a composite of glass plies bonded together by polymeric interlayers—can be used for structural purposes [9,10]. In the event of fracture, the interlayer prevents cracks from propagating through the other plies of laminated glass, stops sharp glass pieces from spreading, and preserves a certain residual load-bearing capacity [11]. In the post-breakage state, the behaviour of laminated glass is influenced by the fragmentation pattern of individual glass panes and the stiffness and strength of the interlayer [12]. For laminated glass, the failure process has been experimentally and numerically investigated for different load conditions and interlayers [13,14]. It has been suggested by Bennison et al. [15] that two main contributions are offered by a fractured layer of glass in a laminated setup: in compression, it can support stresses via the contact between the glass fragments, while in tension, the adhesion between the glass and the interlayer can provide a contribution in terms of tension stiffening of the polymeric film, which would otherwise present negligible stiffness. A discrete element method was employed in [16], in which each element of a broken glass pane was considered to interact with the neighbouring fragments and the interlayer to which it was attached. A similar idea with a combined Voronoi and finite–discrete element method was applied to reconstruct a post-fracture model of laminated glass [17]. Connections between constructional components represent one of the major challenges in glass engineering. Regarding the minimisation of visual impact, bolted connections are the most effective and present the most common fixing techniques for architectural glazing [18]. Due to the discrete size of the bolts, the elements make the fixings almost invisible compared to conventional linear supports. Some examples of point-fixed laminated glass used as canopies above buildings’ entrances are provided in Figure 1. Toughened glass is usually required for point-fixed glazing due to the high stress intensifications close to glass holes [4]. Another reason for using this type of glass in canopies is its increased impact strength compared to annealed float glass [4,19]. Due to the required post-breakage capacity, the use of laminated glass is obligatory. In the case of overhead glass, as shown in Figure 1, the post-breakage performance is particularly critical, since the elements are mounted above the building’s users. In the case of accidental or intentional glass breakage, the element must be sustained at its supports for a defined time so that it does not threaten the building’s users.

Experimental research on the mechanical behaviour of point-fixed laminated glass in an elastic state has been a topic of many studies [20,21,22,23,24]. However, research on post-breakage performance is still limited. This is mainly due to the phenomenon’s complexity and the time-consuming and costly experimental research. Numerical analyses of this phenomenon are highly complex and require advanced materials models; thus, experiments are the only way to assess the post-breakage performance. As reported in [25], point-fixed laminated glass elements tested in the post-breakage state may collapse within minutes due to the tearing of the interlayer at the fixing under certain circumstances (e.g., elevated temperature and a certain amount of sustained load).

Previous research shows that combining glass with other materials has beneficial effects on the post-breakage capacity of the elements [26,27,28,29,30,31]. Meshes and textiles placed in the layer of the foil before the lamination process can also offer enhanced mechanical resistance to ordinary glass structures [32]. In [33,34,35], GFRP composites were used to reinforce the bolted joints in glass specimens subjected to tension. The load-bearing capacity of the strengthened joints was approximately 150% higher than that of the reference specimens. The reinforced joints also showed an ability to resist the applied load after the peak load, i.e., even after the glass failed in the vicinity of the bolt.

The present paper deals with the phenomenon of post-breakage capacity in point-fixed laminated glass elements. This work reports the results of an experimental campaign on the pull-through resistance of laminated glass with locally embedded steel mesh inserts, supported by numerical analyses in the elastic state.

## 2. Materials and Methods

### 2.1. Materials

In this study, a regular soda–lime–silicate float glass was used. This is the most popular type of glass used for architectural applications. Due to the presence of high stresses in glass in the vicinity of holes, fully toughened glass was used. Due to the stress profile introduced in the toughening process, its characteristic tensile bending strength (120 MPa) is over 2–3 times higher than that of annealed float glass. An ethylene vinyl acetate (EVA) interlayer was used for laminating the glass panes [36]. This is an alternative to the commonly used polyvinyl butyral (PVB) interlayer. It has been commonly used for photovoltaic modules, but in recent years it has also attracted attention for architectural applications due to its low production temperature and the fact that it does not require an autoclave in the production process, making it a cheaper alternative to the standard PVB interlayer. EVA also allows distinctive products—such as fabrics, paper, decorative wire mesh, printed PET films, and photovoltaic cells (solar panels)—to be combined in the glass build-up during the lamination process. Moreover, EVA shows better moisture resistance and less delamination probability than PVB. All steel elements of the test setup were made of S355-grade steel [37]. The 12 mm diameter threaded rods were made of 8.8 class bolts, resulting in a tensile resistance of 48.6 kN—much higher than the capacity of samples assessed in the preliminary studies. To avoid stress concentrations between the steel components and glass that could lead to premature glass fracture, 6 mm thick polytetrafluoroethylene (PTFE) spacers were used.

### 2.2. Test Specimens and Test Setup

**Figure 2:**
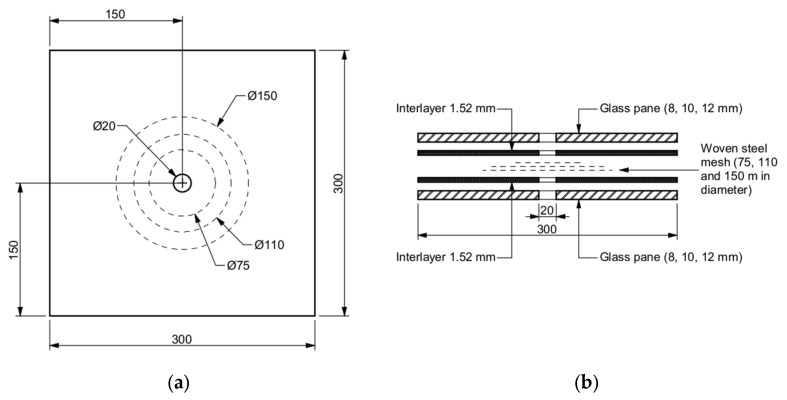
Produced samples: (**a**) plan view; (**b**) exploded view. Dimensions in mm.

In the experimental campaign, laminated glass elements were used (Figure 2). The specimens had dimensions of 300 × 300 mm^2^ and were composed of two toughened glass panes (8, 10, and 12 mm in thickness) and a 3.04 mm thick EVA interlayer. It should be emphasised that the thickness of the interlayer was greater than in conventional build-ups. The reason for this was the requirements of the production process of laminating inserts in the interlayer based on trial samples. This resulted in the use of a greater thickness of the interlayer in order to properly perform the lamination process. A target selection of glass build-up was made based on the size and the type of glazing commonly used in architectural applications. In the centre of each specimen, a through-hole with a diameter of 20 mm was produced by the waterjet technique.

Unreinforced samples (REF) consisted only of glass panes and the interlayer, while in the case of reinforced samples (R), a woven stainless steel mesh of different diameters was placed between two layers of the interlayer before the lamination process. The steel mesh consisted of wires (0.35 mm in diameter) at a spacing of 1 × 1 mm^2^. For further validation of the numerical models, strain gauges were mounted at 55 mm from the hole’s edge on selected samples. Three different pane thicknesses and three diameters of reinforcement were considered in this study. This led to a total of 12 test series and 48 specimens (see Table 1).

### 2.3. Experiments

#### 2.3.1. Monotonic Loading

**Figure 3:**
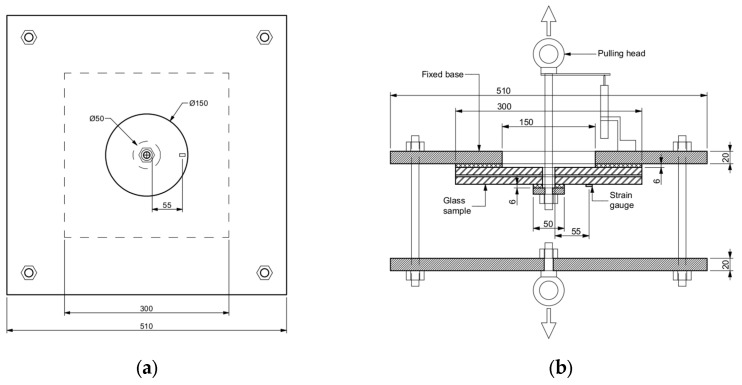
Experimental set-up: (**a**) plan view; (**b**) cross-section. Dimensions in mm.

The setup of the pull-through resistance experiment for laminated glass is shown in Figure 3. Since no standard method for the determination of the mechanical resistance of the connection between the laminated glass and the point-fixing exists, the experimental methodology was inspired by the European Assessment Document (EAD) 090062-00-0404 [38], which covers the assessment of kits for mechanically fixed external wall claddings. The experimental setup included the pulling head and the fixed base, both made of S355 steel. The vertically restrained base was composed of two 510 × 510 mm^2^ steel plates, both 20 mm in thickness, connected by four bolts at the corners, which were 12 mm in diameter. The bottom plate had a central hole (20 mm in diameter) for an eyebolt to be mounted to the lower grip fixture at the load frame of the testing machine. The upper plate of the fixed base had a centrally located hole with a diameter of 150 mm for the puling head to go through with sufficient clearance. The hole diameter was defined based on a preliminary numerical study and literature review. The pulling head consisted of a threaded rod 12 mm in diameter, an eyebolt to connect to the cross-head of the machine, and a steel disk plate (10 mm thick and 50 mm in diameter) to introduce lateral loading to the specimen. During the experiments, the pulling head was used to apply the displacement. Prior to testing, the specimens were placed between the pulling head and the fixed base of the experimental setup. To evenly distribute the load and avoid local stress concentrations in the glass, 6 mm thick PTFE spacers were placed between the tested specimen and the steel plates. The experiment on the pull-through resistance of laminated glass was carried out on a displacement-controlled tensile testing machine with a capacity of 300 kN. The tests were performed at a relative humidity of 50% and room temperature. Several static quantities were monitored during the testing. The signals from the load cell and the cross-head displacement were recorded and sent to an external data collection device. Moreover, a linear variable differential transformer (LVDT) with a measuring length of ±10 mm was mounted to the upper plate of the fixed base and used to measure the pulling head’s local displacement. A strain gauge was connected to the system to measure strain at the surface of the selected samples. All signals were collected on the external data collection device with a data acquisition of 100 Hz.

#### 2.3.2. Creep Test

A creep experiment was performed to study the mechanical behaviour of the pull-through resistance and post-breakage strength of laminated glass under long-term loads. This was essential because the interlayer shows highly viscoelastic behaviour. The experiments were carried out in the same setup as the monotonic loading. Initially, the panes of the specimen were deliberately fractured by hitting their edges with a chisel and a hammer. Subsequently, the sample was placed in the experimental fixture, and a constant force was applied to the pulling head. To reach the target force, the maximum speed of the test machine (100 mm/min) was applied. The creep test monitored the increase in vertical displacement over time. The aim of the experiment was a qualitative assessment of the creep capacity and evaluation of the effectiveness of the embedded steel mesh in terms of long-term behaviour in the post-fractured state. Due to practical concerns and the limited number of samples, only one repetition of the creep test was performed for each test setup.

### 2.4. Numerical Modelling

A three-dimensional numerical model was developed using the commercial FE analysis software ABAQUS/Standard 2020 by Dassault Systèmes (Vélizy-Villacoublay, France) [39]. The model represented the geometric and mechanical properties of the test setup. One-quarter of the nominal geometry with adequate boundary conditions was modelled to reduce the number of FEs and increase the computational efficiency of the simulations.

**Figure 4:**
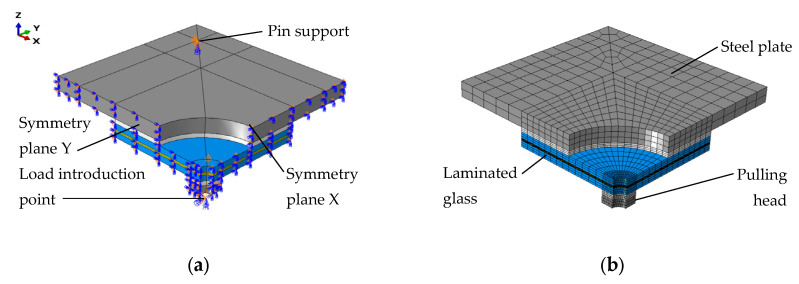
Numerical model: (**a**) boundary conditions; (**b**) mesh pattern.

A numerical reference model is shown in Figure 4a. It consists of a large steel plate representing the fixed base of the experimental setup (in grey) and a PTFE spacer (in white) between the plate and the glass specimen. The model includes the laminated glass specimen, which consists of two glass panes (in blue) and an interlayer (in yellow). The pulling head of the experimental fixture is represented by a steel plate and a PTFE spacer.

All elements were modelled with a set of 3D 8-node solid elements with full integration (C3D8 type from the ABAQUS element library [39]). The surface-to-surface contact interactions between the glass sample and the PTFE spacers that allowed lifting and relative sliding with a friction coefficient of 0.1 were assumed [40]. In the case of the interface between the PTFE spacers and the steel parts, a friction coefficient of 0.18 was applied [40]. The common nodes of the glass panes and the interlayer were constrained by tie interaction, so there was no relative motion between them [39].

Since the numerical study aimed to support the interpretation of the experimental results regarding the analysis of stress in glass at fracture, linear elastic material models were applied. The glass material was represented using linear elastic properties with the density ρ = 2500 kg/m^3^, Young’s modulus E = 70 GPa, and Poisson’s ratio ν = 0.23 [41]. The steel material was modelled with the following linear elastic material model properties: ρ = 7850 kg/m^3^, E = 210 GPa, and ν = 0.30 [37]. Regarding the PTFE, based on a four-point bending test of the spacer, it was found that ρ = 2200 kg/m^3^ and E = 1.06 GPa, while ν = 0.46 was taken from the literature [42]. The material properties of the interlayer were E = 4.85 MPa and ν = 0.45 for 5 min of loading [43].

Figure 4a presents the boundary conditions applied in the model. Since the model represents one-quarter of the nominal geometry, symmetry planes were applied to all components’ X and Y surfaces. Additionally, at the bolt connection, pin support was assumed. The loading was applied at the reference point, which was coupled with the lower surface of the small steel plate. The coupling restraint allowed us to transfer only the vertical displacement of the reference point.

Following a mesh sensitivity study aiming at the verification of the mesh quality, a mesh pattern—shown in Figure 4b—was applied. The sensitivity study focused on the relative change of the normalised maximum principal stress in glass to the number of divisions of its edges and through the thickness. It was found that a combination of a fine 3 × 3 mm^2^ mesh with a coarse 10 × 10 mm^2^ mesh is sufficient to approximate stress in glass. Further refinement of the mesh produced results that did not differ by more than 2%.

## 3. Results and Discussion

### 3.1. Monotonic Loading

**Figure 5:**
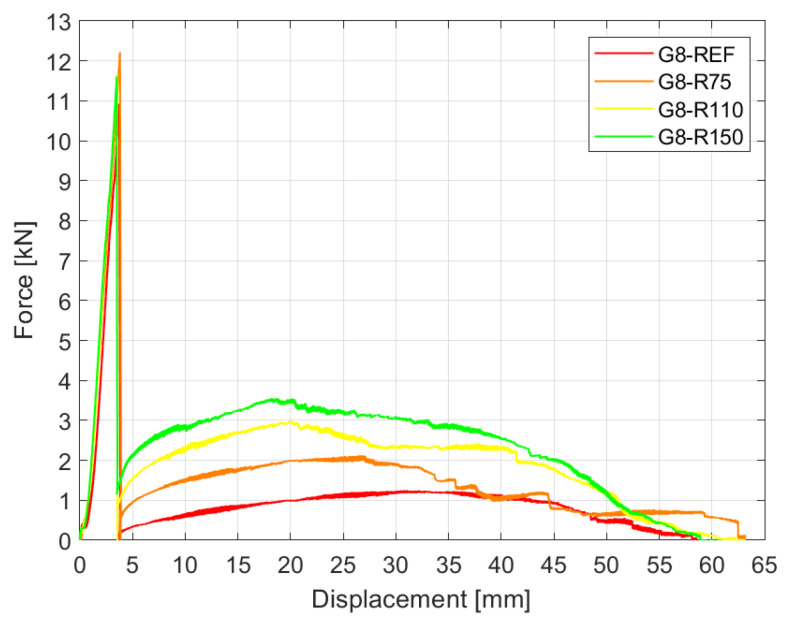
Results of experiments: force–displacement history for test series G8.

Figure 5 presents the exemplary force–displacement history for samples of the glass build-up made of two 8 mm glass plies (test series G8). It compares the results for the reference sample (REF) and reinforced specimens with different diameters of embedded steel mesh (R75, R110, and R150) during monotonic loading. The horizontal axis is based on the data from cross-head displacement.

**Figure 6:**
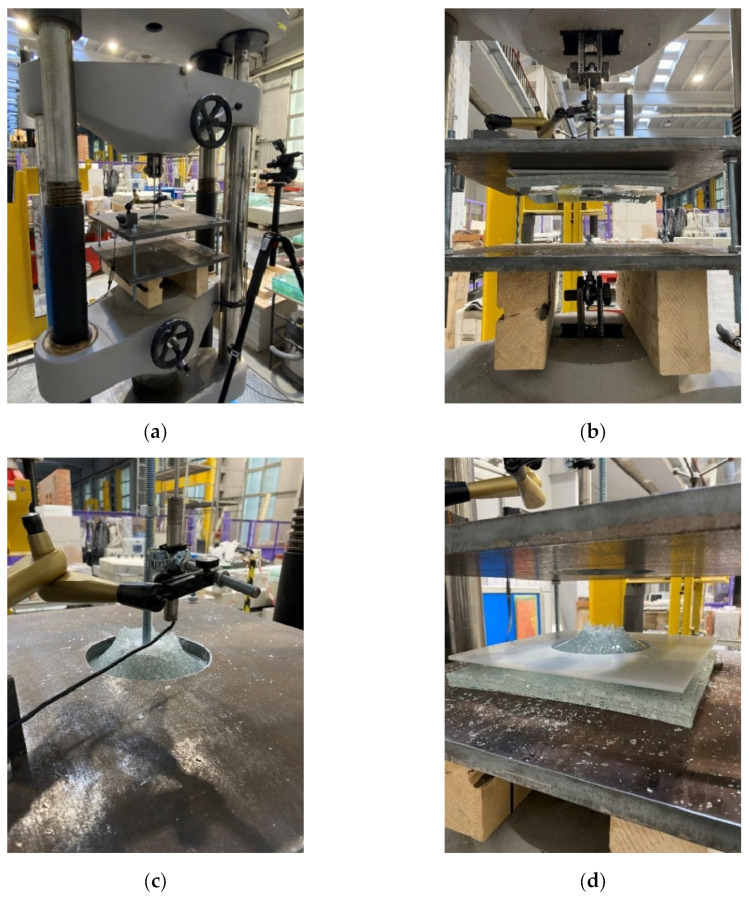
Experiments: (**a**) test setup; (**b**) deformation of the sample in the post-breakage state (view from below the fixed base); (**c**) deformation of the sample in the post-breakage state (view from below the fixed base); (**d**) end of experiment (ultimate failure).

Figure 6 shows the experimental test setup and pictures from the experiments. A few stages of the global mechanism of failure of the samples can be observed. In the first phase, the relationship between the load and cross-head displacement is almost perfectly linear until the moment when the principal stress in the glass reaches its ultimate value. The glass fracture is followed by a sudden drop in the force, which is directly related to the almost complete loss of stiffness (in tension) during fracture of the toughened glass. An instant deformation of the sample in at this stage can be observed (see Figure 6b). In the experiments, two glass panes broke simultaneously for all specimens, indicating that in the event of breakage of a single pane, the other achieves failure stress immediately.

After the glass failure, the interlayer, together with the cracked glass compression zones, allows the sample to carry a limited load; thus, the force does not drop to zero. It is worth noting that different levels of residual force were achieved after glass breakage for the reinforced specimens—the larger the diameter of the reinforcement, the greater the force after glass fracture. In the second stage, the specimen shows post-breakage behaviour, in which the force increases to a certain level and then decreases slowly until the interlayer tears and the puling head of the fixture passes entirely through the sample (see Figure 6c,d). For all specimens, the load increase after glass breakage was observed. However, the reinforced specimens with embedded steel mesh showed improved post-breakage capacity. This clearly indicates the beneficial effect of the steel mesh insert, which smears the local stresses in the interlayer over a larger area and takes on some of the load.

It should be emphasised that the test methodology does not reflect the real situation, where the failure force also acts on the element after glass fracture. In this case, the element would collapse instantly, since the maximum post-critical force is much lower than the load, causing glass failure. This, however, results from displacement-controlled loading, which is a standard method for determination of the post-breakage capacity of building elements and connectors.

**Figure 7:**
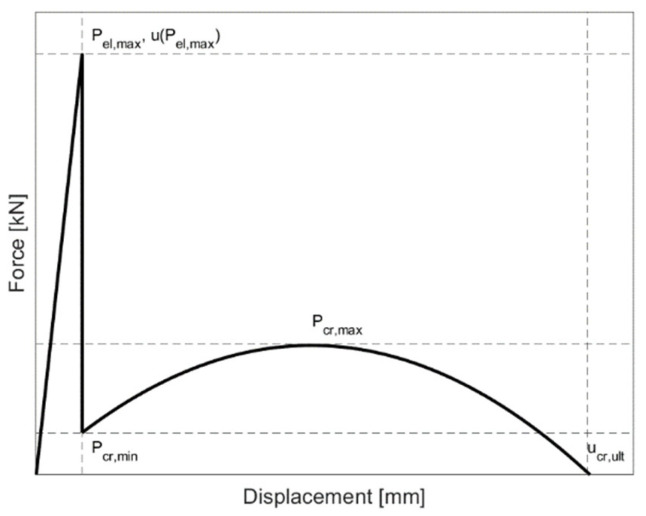
Schematic representation of the global behaviour of the specimens during the experiments.

Figure 7 presents a schematic representation of the specimens’ global behaviour during experiments to evaluate the results quantitatively. The notation el refers to the elastic stage of the behaviour, while the notation cr indicates the post-elastic (cracked) phase.

The average values of the forces and displacements at characteristic phases for all test series, together with the corresponding coefficients of variation, are summarised in Table 2. No difference in the ultimate displacement u_ult_ was observed. All samples failed at the average value of 63.63 ± 0.91 mm. This indicates that the ultimate failure is related to the damage to the interlayer rather than the reinforcing mesh.

It should be noted that in the experiments a relatively high average force (P_el,max_) was obtained at glass fracture, exceeding the load capacity of a typical glass point-fixing. However, the observed value was related to the test configuration and the ratio of the thickness to the span of the specimen.

**Figure 8:**
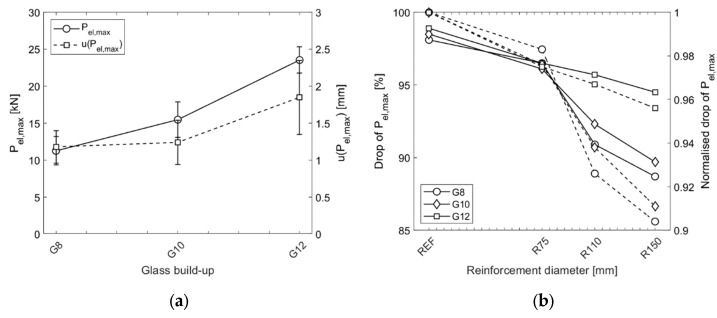
Results of experiments: (**a**) average force and displacement at glass breakage; (**b**) drop in P_el,max_ force after glass breakage (normalised values are marked with dashed lines).

Figure 8a presents the average force at glass breakage P_el,max_ and the corresponding displacement u(P_el,max_) obtained from the experiments. The results were not split into groups with different diameters of reinforcement because the negligible bending stiffness of the steel meshes did not contribute to the specimens’ mechanical (bending) capacity in the elastic stage. An apparent increase in the force at glass breakage was observed with the thicker glasses. The average values of P_el,max_ were 11.25, 15.47, and 23.54 kN and those of u(P_el,max_) were 1.18, 1.24, and 1.85 mm for the specimens G8, G10, and G12, respectively. It should be noted that these values correspond to the local measurements u(P_el,max_) obtained with the LVDT, disregarding the test setup’s deformation. The average variation of the P_el,max_ being 13.4% may seem high; however, this is typical for brittle materials such as glass and is consistent with the results of the experiments reported in [44]. Figure 8b presents the drop in absolute values and those normalised to the reference value (i.e., the average drop for REF samples) as a ratio of P_el,max_ to P_cr,min_. In general, for the reference samples (REF) and the smallest diameter of reinforcement (R75), the drop values were similar, while for R110 and R150 they were considerably smaller. Moreover, in the case of G8 samples (with 8 mm glass), the relationship between the drop in P_el,max_ after glass breakage and the increasing diameter of the reinforcement (for all diameters) was linear. For series G10 and G12 (samples with glass thicknesses of 10 and 12 mm, respectively), the drop value was considerably lower. One possible explanation for this is the fact that after glass fracture, a negligible area of the reinforcement for R75 was involved in resisting the loading, while for R110 and R150 the reinforcement was located in the part of the sample that was subjected to tensile stress.

**Figure 9:**
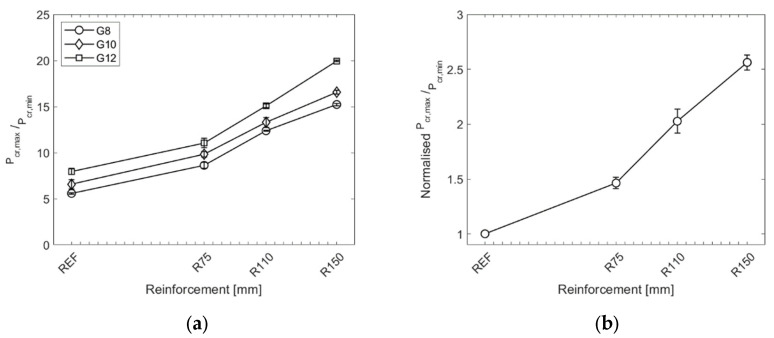
Results of experiments: (**a**) average ratio of P_el,max_ to P_cr,min,REF_ for all specimens; (**b**) normalised values of the ratio P_el,max_/P_cr,min,REF_ for all specimens.

The last step of assessing the experimental results was to evaluate the local reinforcement’s effectiveness on the tested samples’ post-breakage capacity. Firstly, the average ratio of P_el,max_ to P_cr,min,REF_ (an average value for the reference specimens) was calculated (Figure 9a). Subsequently, the ratios were averaged among the glass build-ups and normalised to the results of the reference (unreinforced) samples. Figure 9b shows the final ratios of normalised P_el,max_/P_cr,min,REF_ for all specimens. It was found that the local reinforcement in the direct vicinity of the hole in the glass increased the post-breakage strength by 46.3%, 102.6%, and 156.2% for the reinforcement diameters of 75 mm, 110 mm, and 150 mm, respectively.

### 3.2. Creep Test

The first step of the study was to determine the target loading in the creep test. To do so, a fractured, unreinforced sample was loaded with a force corresponding to 80% of the *Pcr,max* determined in the monotonic test. Subsequently, the load was gradually increased by 100 N until a significant increase in the displacement rate was observed. In this way, the creep experiment’s target force was determined for all test series. The exact target force was applied to the reference and reinforced samples.

**Figure 10:**
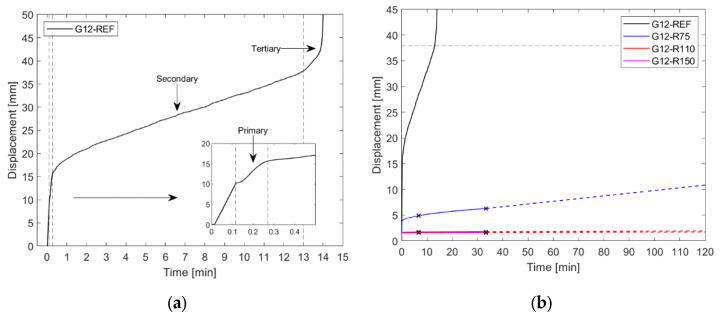
Results of creep experiments: (**a**) exemplary time–displacement history for the reference sample G12 (dashed lines represent delimitation among phases of creep behaviour); (**b**) extrapolated displacement history for test series G12 (dashes, color lines represent extrapolated values based on two points marked with “^x^” symbol).

Figure 10a presents the exemplary time–displacement history for the reference sample G12 subjected to the target force. The creep behaviour can be split into three prominent phases: Primary (transient) creep shows a steady decrease in creep rate. Dislocations are formed in the material during limited plastic deformation, and dislocation hardening occurs. Secondary creep takes place at an approximately constant creep rate. The dislocation density is now sufficient, and annihilation of dislocations takes place. The annihilation effect can be described as a softening of the material. Finally, tertiary creep occurs, eventually causing the material to rupture. Voids are now present in the grain boundaries, weakening the material and increasing the creep rate. Figure 10b shows the results for all specimens within the G12 test series. For the purpose of clarity, the graph is limited to 120 min. Due to time constraints in the laboratory, the force was only held for the time corresponding to triple the time at failure for the reference sample G12-REF. The sample behaviour was then analytically extrapolated (in time) until the same failure displacement was achieved as for the reference sample. This approach seems justified since, in the monotonic tests, all samples (reference and reinforced) failed at the same displacement.

Table 3 presents the creep results for all test series. This involves a target force applied to the specimen, an instantaneous displacement after its introduction, a measured displacement rate in the secondary creep phase, and estimated time to failure based on the extrapolated time–displacement histories.

The qualitative creep assessment shows that the reinforcing mesh significantly increases the time to failure. This phenomenon is especially evident with the smallest mesh, when the G75 specimens increase the time to failure by an average factor of 6.5. This can be explained by the fact that the reinforcement decreases the local stress in the interlayer which, in the case of unreinforced samples, carries the entire loading in the post-breakage state. It should be emphasised that the beneficial effect of the reinforcement on the creep behaviour was clearly affected by the applied thickness of the interlayer which, due to the production requirements, was thicker than in standard applications.

**Figure 11:**
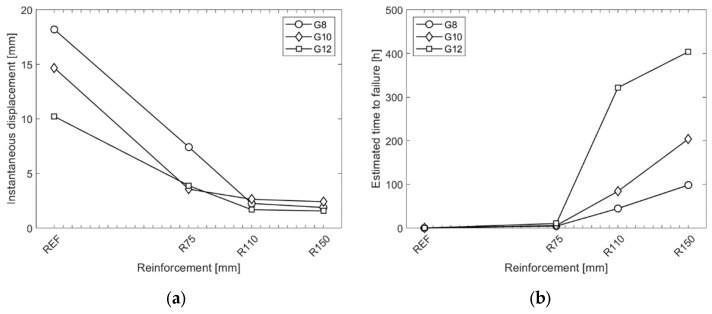
Results of creep experiments: (**a**) instantaneous displacement after constant force; (**b**) estimated time to failure.

Figure 11a,b summarise the results obtained from the creep experiments and present the instantaneous displacement of the samples under the target load and the estimated time to failure for the reference and reinforced samples, respectively. It is evident that the reinforcement improves the creep behaviour of the samples, leading to less instantaneous deformation under constant loading and a reduced displacement rate. Since the reinforcement reduces the stresses in the interlayer and takes on some of the load, it significantly extends the time until failure.

### 3.3. Numerical Modelling

**Figure 12:**
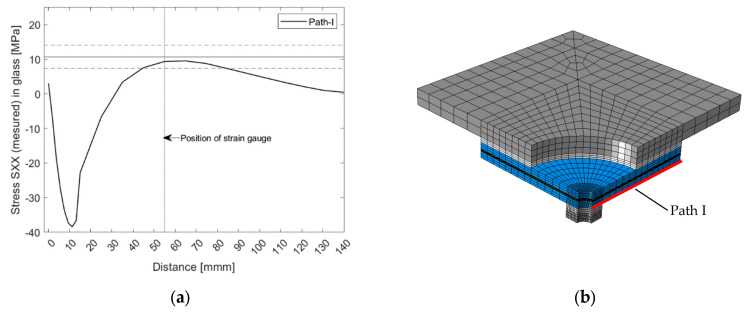
Results of the numerical study for test series G8: (**a**) stress SXX profile along Path-I; (**b**) location of Path-I.

The analysis aimed to determine the stress in the glass at initial fracture. This is critical for further studies regarding the development of a constitutive model for cracked glass that are beyond the scope of the present paper. The model of G8 specimen was first validated by the experimental results (i.e., strains measured by a strain gauge mounted on glass, as shown in Figure 3). Figure 12a shows stress SXX along the path at the glass edge on the puling head side (Figure 12b). In addition, the position of the strain gauge and the average value (together with the upper and lower bounds) of the stress were measured during the experiments. The numerical model simulated the experiments reasonably well; the simulated stress at the gauge position was approximately 12% lower than the measured value.

**Figure 13:**
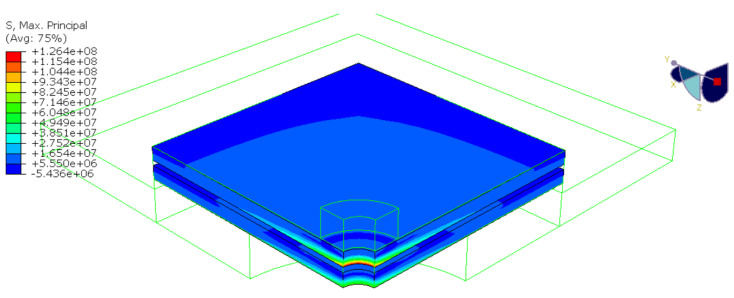
Results of the numerical study for test series G8: maximum principal (tensile) stress in glass at failure (values in Pa).

Figure 13 presents the principal (tensile) stress in glass at the same displacement corresponding to the average failure load. The maximum principal stress occurred at the glass hole in the pane from the pulling head side. Its value was much higher than the value of stress in the X direction since it included radial and circumferential stress components. The validated model was then used to simulate the stress in other samples (i.e., G10 and G12). The analyses found that the average value of principal (tensile) stress at failure for all specimens was 171.7 ± 36.4 MPa.

## 4. Conclusions

This article presents the results of experimental studies on the pull-through resistance of reinforced point-fixed laminated glass elements with locally embedded steel mesh. It involved monotonic and creep studies in a custom-made experimental setup. Numerical simulations were performed to determine the failure stress in glass. The following conclusions can be drawn from the conducted research:

- Regarding the monotonic loading experiment, the load increase after glass breakage was observed for all specimens; however, the reinforced specimens with embedded steel mesh showed significantly improved post-breakage capacity. It was found that the local reinforcement in the direct vicinity of the hole in glass increased the post-breakage strength by 46.3%, 102.6%, and 156.2% for the reinforcement diameters of 75 mm, 110 mm, and 150 mm, respectively. All samples failed at the average value of 63.63 ± 0.91 mm, indicating that the ultimate failure is related to the damage to the interlayer rather than the reinforcing mesh.
- The creep study found that the reinforcing mesh significantly increased the time to failure. This was especially valid for the smallest mesh (75 mm in diameter), in which the time to failure was increased by a factor of 6.5.

It should be emphasised that the above conclusions are valid only for the configuration of test conditions used in the study. The phenomenon of the post-breakage resistance of laminated glass is highly complex and requires further studies, including investigations of varying build-up configurations under different temperatures.

## Figures and Tables

**Table 1:** Overview of the produced specimens. In the test series, “G” stands for glass build-up, while “R” indicates the diameter of the steel mesh in the reinforced samples.

Test Series	Glass Thickness (mm)	Interlayer	Reinforcement
G8-REF	2 × 8 mm	2 × 1.52 mm	-
G8-R75			Ø75 mm
G8-R110			Ø110 mm
G8-R150			Ø150 mm
G10-REF	2 × 10 mm		-
G10-R75			Ø75 mm
G10-R110			Ø110 mm
G10-R150			Ø150 mm
G12-REF	2 × 12 mm		-
G12-R75			Ø75 mm
G12-R110			Ø110 mm
G12-R150			Ø150 mm

**Table 2:** Results of the experimental study.

Test Series	P_el,max_ (kN)	u(P_el,max_) * (mm)	P_cr,min_ (kN)	P_cr,max_ (kN)	u_ult_ (mm)
G8-REF	11.86 ± 1.62	1.50 ± 0.05	0.22 ± 0.08	1.39 ± 0.06	63.30 ± 3.09
G8-R75	11.59 ± 1.71	1.09 ± 0.13	0.41 ± 0.16	2.10 ± 0.35	60.48 ± 4.58
G8-R110	10.09 ± 2.46	0.95 ± 0.10	0.92 ± 0.12	3.01 ± 0.07	65.08 ± 0.94
G8-R150	11.48 ± 1.10	1.22 ± 0.13	1.30 ± 0.06	3.70 ± 0.13	63.30 ± 2.23
G10-REF	17.38 ± 2.27	1.31 ± 0.22	0.27 ± 0.10	1.60 ± 0.04	64.33 ± 1.33
G10-R75	16.42 ± 1.45	1.19 ± 0.33	0.65 ± 0.06	2.39 ± 0.31	59.93 ± 4.19
G10-R110	13.14 ± 2.08	1.22 ± 0.37	1.01 ± 0.19	3.23 ± 0.53	66.58 ± 0.11
G10-R150	14.92 ± 1.20	1.22 ± 0.24	1.52 ± 0.27	4.03 ± 0.21	61.19 ± 1.52
G12-REF	22.34 ± 2.05	2.12 ± 0.79	0.24 ± 0.08	1.93 ± 0.33	65.01 ± 2.47
G12-R75	23.91 ± 0.88	1.67 ± 0.27	0.85 ± 0.11	2.68 ± 0.50	53.40 ± 6.67
G12-R110	23.33 ± 1.29	1.75 ± 0.35	1.01 ± 0.55	3.67 ± 0.21	66.64 ± 0.05
G12-R150	24.57 ± 1.82	1.87 ± 0.27	1.34 ± 0.66	4.85 ± 0.08	65.63 ± 0.91

* Based on local measurements (LVDT).

**Table 3:** Results of creep experiment.

Test Series	Constant Force (kN)	Instantaneous Displacement (mm)	Displacement Rate (mm/min)	Estimated Time to Failure (h)
G8-REF	1.1	18.20	1.6402	0.13
G8-R75	1.1	7.42	0.1062	4.65
G8-R110	1.1	2.26	0.0139	44.95
G8-R150	1.1	1.89	0.0064	98.64
G10-REF	1.3	14.68	2.3687	0.09
G10-R75	1.3	3.57	0.0855	5.92
G10-R110	1.3	2.64	0.0065	84.39
G10-R150	1.3	2.42	0.0027	203.98
G12-REF	1.4	10.23	1.4761	0.22
G12-R75	1.4	3.89	0.0525	10.59
G12-R110	1.4	1.69	0.0019	321.70
G12-R150	1.4	1.57	0.0015	403.77

## Data Availability

Not applicable.
